# Beta-glucans in advanced CKD: role in endotoxaemia and inflammation

**DOI:** 10.1186/s12882-020-01779-9

**Published:** 2020-04-06

**Authors:** Jonathan Wong, Yonglong Zhang, Oscar Swift, Malcolm Finkelman, Ashish Patidar, Sivaramakrishnan Ramanarayanan, Enric Vilar, Ken Farrington

**Affiliations:** 1 Renal Research, East and North Herts NHS Trust, Coreys Mill Lane, Stevenage, SG1 4AB UK; 2 University of Hertfordshire, Hatfield, UK; 3 Associates of Cape Cod Inc, Massachusetts, UK

**Keywords:** Endotoxin, (1–3)-β-D glucan, Inflammation, Dialysis

## Abstract

**Background/aims:**

(1–3)-β-D glucans (BG) are cellular components of yeasts and fungi. Elevated blood levels may be an adjunct in diagnosing invasive fungal infection, though can be high in dialysis patients without fungaemia. BG can also induce false positive signals in endotoxin detection assays (Limulus Amoebocyte Lysate [LAL] assay). We explored the relationship between BG levels, renal impairment, endotoxaemia and inflammation.

**Methods:**

We measured serum BG levels, markers of inflammation and blood endotoxin levels in 20 controls, 20 with stages 1–3 chronic kidney disease (CKD), 20 with stages 4–5 CKD, 15 on peritoneal dialysis (PD) and 60 on haemodialysis (HD). Another 30 patients were studied before and after HD initiation.

**Results:**

BG levels increased with advancing CKD, being highest in HD patients, 22% of whom had elevated levels (> 80 pg/ml). Levels increased significantly following HD initiation. Levels also correlated positively with CRP, TNFα, IL-6 levels, independently of CKD stage. Blood endotoxin was detectable by LAL assays in 10–53% of the CKD cohort, being most prevalent in the HD group, and correlating positively with BG levels. Adding BG blocking agent to the assay reduced endotoxin detection confining it to only 5% of HD patients. Levels of inflammatory markers were higher in those with detectable endotoxin - whether false- or true positives.

**Conclusion:**

BG levels increased with decreasing renal function, being highest in dialysis patients. High BG levels were associated with false positive blood endotoxin signals, and with markers of inflammation, independently of CKD stage. The cause for high BG levels is unknown but could reflect increased gut permeability and altered mononuclear phagocytic system function.

## Background

Beta glucans [(1–3)-β-D glucans] (BG) are major carbohydrate constituents of the cell walls of yeast and fungi. Recently, they have attracted significant scientific attention because of their reported biological activities, which include anticancer, anti-inflammatory and immune-modulating effects [1]. However, BGs are also key pathogen-associated molecular pattern molecules that trigger a number of host immune responses and can stimulate the production of reactive oxygen species and inflammatory cytokines mediated by interaction with dectin-1 and toll-like receptors [2]. Elevated blood BG levels have been used as a diagnostic adjunct for invasive fungal infections and an assay has been developed and cleared by the US Food and Drug Administration for this purpose [3].

In haemodialysis (HD) patients, high blood levels of BG can activate blood endotoxin detection assays such as the Limulus Amoebocyte Lysate (LAL) assay leading to apparently increased blood endotoxin levels [4]. False positive signals for endotoxemia occurred in 50% of HD patients which were extinguished on remeasurement incorporating a BG-blocking agent with the LAL assay [5]. BG levels have been found to be elevated both in patients on HD and in those on peritoneal dialysis (PD) [6, 7]. Dialysis membranes can increase BG levels but the effect appears to be confined to now obsolete cellulose membranes and this phenomenon is not seen with modern biocompatible devices [8–10]. BG is cleared via the reticuloendothelial system and clearance is thought to be independent of renal function [11, 12]. Hence the cause for elevated BG levels is not clear.

The purpose of this study was to explore the prevalence and clinical consequences of elevated BG levels in non-infected subjects with CKD. We aimed to study BG levels in patients across the spectrum of CKD to investigate their associations with detectable blood endotoxin levels and markers of inflammation.

## Methods

### Design and setting

This was a single centre study conducted at the East and North Hertfordshire NHS Trust. Inclusion criteria were adults aged 18 years and over. Participants consisted of healthy controls, non-dialysed CKD, PD and HD patients. Exclusion criteria include participants with active sepsis, positive HIV, hepatitis B or C serology, pregnancy and those with active vasculitis or connective tissue disease.

### Study design

#### Sub-study 1

The study aimed to investigate BG levels in healthy controls, CKD and dialysis patients. Across this spectrum, the associations of BG levels with markers of inflammation and endotoxin levels, were explored. 135 subjects were recruited (20 healthy controls, 20 patients with CKD 1–3 (eGFR≥30 mL/min), 20 patients with CKD 4–5 (eGFR< 30 mL/min), 15 PD patients and 60 HD patients). Patients with CKD were recruited from nephrology clinics at our trust, all patients had a confirmed diagnosis of CKD based on reduced estimated GFR with either structural, histological evidence or had significant proteinuria. Estimated GFR was calculated using the Modification of Diet in Renal Disease (MDRD) study. The HD group consisted of two equal subgroups, a group chosen considered to be at high risk of endotoxemia, and a group whose risk was considered lower. Patient considered to be high-risk had chronic unexplained inflammation indicated by a raised CRP > 5 mg/L, measured on two separate occasions at least 1 month apart during the 3 months prior to study recruitment together with either a high ultrafiltration requirement (rate > 10 mL/kg/hr) (11) or pre- or post-dialysis systolic BP < 100 mmHg within the week prior to study recruitment. Patients with high ultrafiltration requirements or low blood pressure are at risk of intra-dialytic hypotension and may predispose to endotoxemia driven by gut hypoperfusion [13–15]. Low-risk patients had none of these features. Patients were assessed to ensure no evidence of sepsis, no recent antibiotic treatment or receipt of blood products in the last month since these can elevate blood BG levels [16].

Blood samples were drawn from all these subjects and measured for BG, endotoxin, IL-6 (interleukin-6), TNF-α (tumour necrosis factor alfa), and CRP levels. Demographic and relevant clinical data were collected for all subjects.

#### Sub-study 2

This study aimed to investigate the effect of HD initiation on blood levels of BG and endotoxin. Thirty patients with CKD stage 5 who were planned for dialysis were recruited for this sub-study. Blood samples were collected within the 6 months before starting HD and repeated within 6 months of initiation to determine the effect of HD initiation on levels of endotoxin, BG and markers of inflammation.

### Blood sampling and processing

Blood samples were collected peripherally using aseptic technique as previously described [17, 18]. For haemodialysis patients, blood samples were collected pre-dialysis. Blood samples were not collected from haemodialysis catheters to avoid endotoxin contamination. Samples for BG measurements and endotoxin were collected in Terumo Venoject II heparinised tubes (Project KBG). Samples for cytokine measurements were collected in S-monovette Z-gel tubes (Sarsedt).

### Laboratory measurements

#### (1–3)-β-D glucan assay

BG measurements was carried out using the Fungitell® assay (Associates of Cape Cod, Inc.) as per manufacturer’s instructions and as previously described ^5^ [19, 20],. Serum samples were mixed with 20 μL pre-treatment buffer (0.125 M KOH/0.6 M KCl), in microplate wells, and incubated at 37 °C for 10 min. Fungitell reagent, reconstituted in 0.1 M Tris HCl, pH 7.4, was added to sample and standard curve wells (7.8–500 pg/mL, Pachyman). The reactions were read kinetically, at 405 nm minus 490 nm at 37 °C, for 40 min. Vmean values (milliabsorbance units/min) were calculated for standards and samples and sample titres interpolated from the standard curve. Coefficient of variation (CV) for all assays was < 20%. Normal human serum contains low levels of BG, typically 10-40 pg/mL [21]. Levels < 60 pg/mL are interpreted as negative and between 60 and 80 pg/mL as indeterminate. Levels > 80 pg/mL are interpreted positive and in at-risk patients considered as a marker of invasive fungal infection [22].

#### Cytokine measurements and C-reactive protein measurements

Serum was measured for IL-6 and TNF-α using enzyme-linked immunosorbent assays (Human Quantikine ELISA, R&D systems). High-sensitivity CRP was measured using particle enhanced immunoturbidimetric assay (Roche Diagnostics).

#### Endotoxin measurements

Endotoxin was measured twice, once using the standard LAL assay [17] and then repeated with a BG-blocking agent which inhibits the factor G pathway preventing the LAL assay from false positive activation by BG that may be present in the sample [4].

#### Endotoxin assay using standard LAL without BG-blocking buffer

Endotoxin measurements were performed using the kinetic turbidimetric LAL assay (Endosafe KTA2, Charles River Laboratories) as previously described [17]. Plasma samples were diluted 1:10 with 0.1% Tween80 (Merck Chemicals) and heated to 70 °C for 10 min to remove inhibitory factors present in plasma and cooled to room temperature prior to analysis. 100 μL of Endosafe KTA2 reagent was added to each 100 μL sample in microplate wells. The plate was monitored at 340 nm using a Biotek ELx808 absorbance microplate reader with Endoscan-V software (version 4.0; Charles River) with an onset optical density of 0.03. Six-point standard curves were constructed using standard dilutions of control standard endotoxin (*E*.*coli* 055:B5) ranging from 10 to 0.0025 EU/mL All standard curves had a correlation coefficient > 0.98.

#### Endotoxin assay using LAL reconstituted with BG-blocking buffer

To prevent false activation of the LAL assay by BG in the sample, Endosafe KTA2 reagent was reconstituted with 5.2 mL BG-blocking agent (Charles River ES-Buffer) containing 1 mg/mL carboxymethylated curdlan [4]. Endotoxin measurements were carried out using the same procedure as described above.

#### Statistical analysis

Analyses were performed using IBM SPSS statistics version 21. Parametric data were presented as mean ± standard deviation. Non-parametric data were presented as median (interquartile range). Comparisons of continuous data between multiple groups used one-way ANOVA or the Kruskal-Wallis test and significance testing was adjusted using Bonferroni correction. Comparison of two groups used t-test or Mann-Whitney U. Proportions were compared using the Chi-squared test. Correlation analyses was performed using Spearman rank correlation coefficient. Logarithmic transformation of variables was used as required to allow the assumptions and conditions for multiple regression to be met. Kaplan-Meier analysis and Cox proportional hazard models were used to explore the relationship between baseline BG levels and outcomes. Follow-up during the study period was complete. Wilcoxon signed rank test was used to compare pre- and post HD initiation levels of BG and other variables.

## Results

### Demographic, clinical and biochemical characteristics

In general patients with advanced CKD and those on dialysis were older and more highly comorbid than those with lesser degrees of CKD and controls (Table 1). PD patients had lower dialysis vintage and higher residual renal function (KRU) than HD patients. Low- and high-risk HD patients (Table 2) were similar with respect to age, co-morbidity and dialysis catheter use. There was a trend towards greater use of haemodiafiltration (HDF) in low-risk HD patients who also had lower dialysis vintage, higher blood pressure, greater KRU, shorter dialysis sessions and higher standard Kt/V. Ultrafiltration rates, phosphate and albumin levels were lower in low risk HD patients but these differences were not statistically significant.

**Table 1:** Patient characteristics and results of investigations Data represent median (interquartile ranges) or mean ± standard deviation according to distribution

	Healthy Control	CKD 1–3	CKD 4–5	PD	Low-risk HD	High-risk HD	***p***-value*
**Age (years)**	48 ± 8	50 ± 20	62 ± 12	60 ± 17	67 ± 17	61 ± 14	**< 0.001**
**Weight (kg)**	74 ± 14	83 ± 20	95 ± 18	85 ± 14	74 ± 19	82 ± 23	**0.005**
**urine protein creatinine ratio (mg/mmoL)**		41 [139]	97 [155]				
^*^GFR/KRU(ml/min)	–	54.4[17.3]	14.2[5.1]	4.1[4.9]	1.6[2.7]	0[0]	**–**
**Anuric (%)**	–	–	–	6.7	33.3	80	**< 0.001**
**Charlson Co-morbidity Index**	–	1 [0–4]	5 [4–8]	6[3–7]	7[4.8–9]	5.5[3.8–8]	**< 0.001**
**Diabetes (%)**	–	25	45	27	40	33	NS
**Dialysis vintage (years)**	–	–	–	1 [0.3–4]	1.6 [0.9–4.3]	3[1.3–6.1]	**0.056**
**BG (pg/mL)**	13.5 [6.5]	15.5 [16]	22.5 [10.5]	37 [36]	55.5 [39]	57 [34.5]	**< 0.001**
**CRP (mg/L)**	1.5 [2.8]	2.9 [7.9]	3.9 [3.8]	4.1 [9.2]	1.9[3.0]	12.9 [11.8]	**< 0.001**
**IL-6 (pg/mL)**	0 [0.8]	3.1 [6.7]	2.4 [5.6]	6.3 [7.9]	8.6 [5.6]	14.3 [13.2]	**< 0.001**
**TNF-α (pg/mL)**	8 [4.1]	7.6 [7.0]	16 [6.6]	15.9 [6.5]	22.4 [8.0]	23.9 [7.0]	**< 0.001**
**Endotoxin level (EU/ml)** **[− BG blockade] (%)**	0	0 [0]	0 [0]	0 [0.049]	0 [0.036]	0.027 [0.041]	**0.001**
**Detectable endotoxin** **[− BG blockade] (%)**	0	10	15	47	37	53	**< 0.001**
**Detectable endotoxin** **[+ BG blockade] (%)**	0	0	0	0	3.3	6.7	NS

eGFR - estimated glomerular filtration rate - applies to healthy and non-dialysed CKD patients, KRU - residual urea clearance - applies to dialysis patients. NS – *p* > 0.1. For other abbreviations see text. Reference ranges for CRP, IL-6 and TNFα are < 5 mg/L, < 3.1 pg/mL and < 15.6 pg/mL respectively. + and - BG blockade refers to performance of LAL assay with and without use of BG blocking buffer to prevent any false activation by any circulating (1–3)-β-D glucan present in the sample. * *P*-value derived from one way ANOVA, Kruskal-Wallis or Chi-square tests as appropriate and denotes overall significant difference between independent groups. See text for relevant individual group differences as appropriate

**Table 2:** Comparison of Low and High Risk Haemodialysis patients.

	High-risk HD	Low-risk HD	***p***-value
**Age (years)**	61 ± 14	67 ± 17	NS
**Weight (kg)**	82 ± 23	74 ± 19	NS
**KRU (ml/min/1.73 m**^**2**^**)**	0[0]	1.6 [2.7]	**< 0.001**
**Anuric (%)**	80	33.3	**< 0.001**
**Charlson co-morbidity score**	5.5[3.8–8]	7[4.8–9]	NS
**Diabetes (%)**	33	40	NS
**Access with THL (%)**	23	17	NS
**Td (min)**	224 ± 25	206 ± 26	**0.007**
**UFR (ml/kg/min)**	5.9 ± 3.5	4.4 ± 3.2	**0.084**
**HDF (%)**	74	90	**0.095**
**Pre-SBP (mmHg)**	131 ± 29	157 ± 24	**< 0.001**
**Pre-DBP (mmHg)**	68 ± 22	74 ± 14	NS
**Post-SBP (mmHg)**	117 ± 31	137 ± 25	**0.007**
**Post-DBP (mmHg)**	63 ± 18	64 ± 12	NS
**Calcium (mmol/l)**	2.32 ± 0.14	2.34 ± O.13	NS
**Phosphate (mmol/l)**	1.90 ± 0.58	1.66 ± 0.44	**0.079**
**Albumin (g/l)**	40 ± 3	38 ± 3	**0.053**
**PTH (pmol/l)**	41.9 [22.5–83.8]	43.4 [27.3–63.7]	NS
**Standard Kt/V**	2.1 ± 0.3	2.5 ± 0.4	**< 0.001**

KRU – residual renal urea clearance; *THL* Tunnelled Haemodialysis catheter, *Td* sessional dialysis time, *UFR* Ultrafiltration rate, *HDF* haemodiafiltration, *Pre-SBP* pre-dialysis systolic blood pressure, *Pre-DBP* pre-dialysis diastolic blood pressure, *post-SBP* post-dialysis systolic blood pressure, *post-DBP* post-dialysis diastolic blood pressure, *PTH* parathyroid hormone, Kt/V normalised dialysis urea clearance. NS – p > 0.1

### Blood levels of (1 → 3)-β-D glucan

**Fig. 1:**
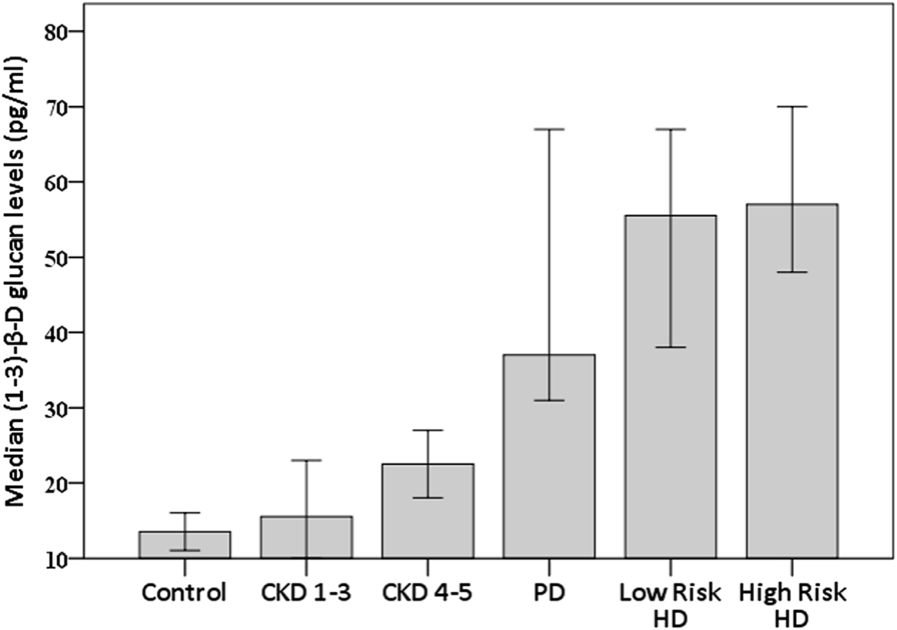
Median (1–3)-β-D glucan levels in controls and at various stages of CKD. Error bars represent 95% confidence limits

BG levels progressively increased with worsening kidney function and BG levels were highest in patients on dialysis [Fig. 1]. High-risk HD patients, low-risk HD patients and PD patients had significantly higher BG levels than CKD 1–3, CKD 4–5 groups and controls (all *p* < 0.005), although there were no significant differences between PD and HD patients. BG levels were not significantly different between high-risk and low-risk HD patients. High BG levels above the cut-off level of 80 pg/mL were found in 22% of HD patients (16.7% low-risk and 26.7% high-risk), 13.3% of PD patients, 0% of CKD 4–5 and 10% of CKD 1–3 patients.

Across the whole CKD group BG correlated with Charlson co-morbidity score (rho = 0.305, *p* = 0.001). There was no relationship to age or gender. In HD patients, BG levels were inversely correlated with KRU (rho = − 0.414, p = 0.001). Levels were similar in those receiving HDF and those on high-flux HD (57 vs. 45 pg/ml, *p* = 0.151). There was no relationship between BG levels and Kt/V.

### Association of blood (1 → 3)-β-D glucan levels with endotoxemia

**Fig. 2:**
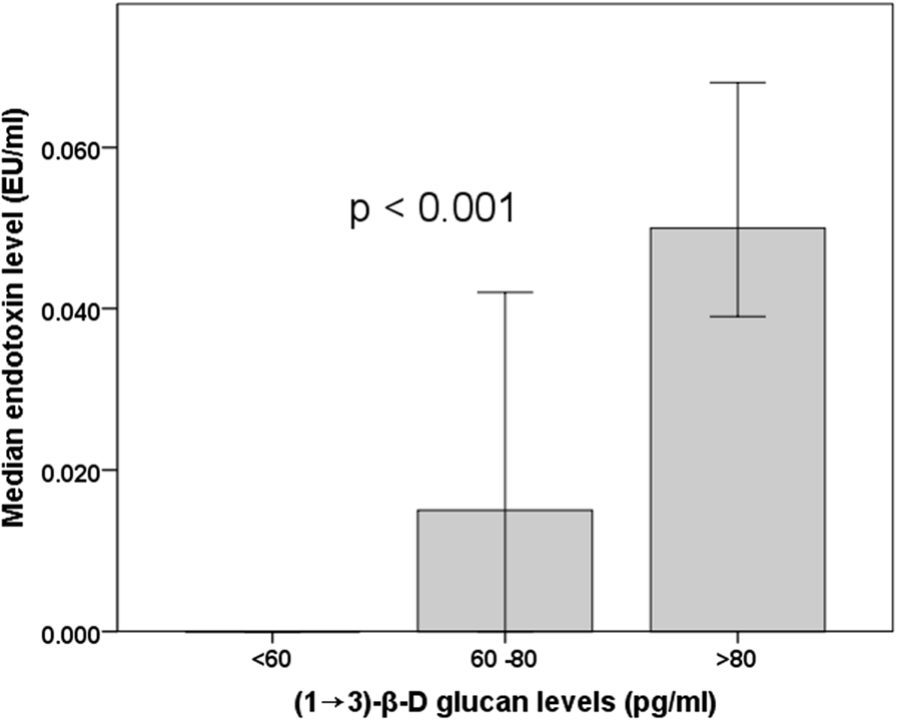
Endotoxin levels in CKD cohort detected using LAL assay without betaglucan blocker stratified according to betaglucan levels. There is a significant difference in endotoxin levels across the three betaglucan groups (*p* < 0.001 by Kruskal-Wallis). Error bars represent 95% confidence limits

Using the standard LAL assay revealed low level blood endotoxin signals in HD, PD and CKD patients (Table 1). Following repeat measurement with a BG-blocking agent, the majority of endotoxin signals were extinguished and only three HD patients in the whole cohort (two high-risk and one low-risk) remained positive for endotoxin. Endotoxin signals derived from the LAL assay without BG blocking agent correlated strongly with BG levels in the CKD cohort (rho = 0.519, *p* < 0.001). Endotoxin signal was highest in subjects with high BG (> 80 pg/mL), intermediate at BG levels 60–80 pg/ml and lowest at low levels (< 60 pg/mL) (p < 0.001) [Fig. 2]. These findings strongly suggest that endotoxin signal detected using the LAL assay in the majority of CKD patients may be artefactual due to elevated BG.

### Associations of blood (1 → 3)-β-D glucan levels with markers of inflammation

High–risk HD patients had higher levels of CRP (*p* < 0.05 for all) than all other groups as expected (Table 1). IL-6 in high-risk HD patients were similar to low-risk HD patients but higher than all other sub-groups (p < 0.05). IL-6 levels in PD patients were significantly higher than controls but similar to CKD 1–3 and CKD 4–5 patients. There was no significant difference in IL-6 between CKD 1–3, CKD 4–5 and healthy controls. For TNF-α, high-risk HD patients were similar to PD and low-risk HD patients but higher than CKD patients and healthy controls. TNF-α in low-risk HD patients were similar to CKD 4–5 patients but higher than CKD 1–3 and healthy controls. PD patient had higher TNF-α than controls but were similar to CKD 1–3 and CKD 4–5 patients. TNF-α was higher in CKD 4–5 compared to CKD 1–3 and controls. Levels of TNF-α were similar between CKD 1–3 and controls.

Among the CKD cohorts, levels of BG correlated significantly with markers of inflammation - CRP (rho = 0.271; *p* = 0.003), IL-6 (rho = 0.520; *p* < 0.001) and TNF-α (rho = 0.486; p < 0.001). In separate models we controlled for CKD group [CKD 1–3, CKD 4–5, PD, and HD]. In these models log BG was an independent predictor of log IL-6 (standardised beta 0.209: *p* = 0.038), log TNF (standardised beta 0.202: *p* = 0.023) and log CRP (standardised beta 0.357: *p* = 0.004).

### Association of endotoxaemia with markers of inflammation

Table 3 depicts the relationship between endotoxin detection and markers of inflammation. In general levels of inflammatory markers were greater in patients in whom endotoxin was detected – whether as a false positive or true endotoxaemia although this was only significant for IL-6. No differences in the levels of inflammatory markers could be detected between those with true endotoxaemia and those with false positive tests but comparisons were hampered by the small number with true endotoxaemia.

**Table 3:** Median levels of inflammatory markers in whole CKD group in relation to endotoxin detection – no endotoxin, false positive endotoxin and true endotoxaemia

Endotoxaemia	None ***n*** = 76	False + ve ***n*** = 36	True ***n*** = 3	***p***-value
**IL-6 (pg/ml)**	7.4 (8.5)	^a^9.4 (13.9)	4.9 {8.2}	0.020
**TNF (pg/ml)**	18.4 (11.7)	20.6 (9.6)	23.7 {1.4}	0.053
**CRP (mg/l)**	3.9 (7.8)	6.4 (10.8)	4.7 {15.0}	0.294

Values quoted are median (interquartile range) except for true endotoxaemia which are median {range} since the number with this condition was only 3. The values quoted for significance relate to the application of the Kruskal-Wallis test across all three groups. ^a^ relates to the application of the Mann-Witney test to compare medians in the no endotoxin and false positive endotoxin groups (*p* = 0.012)

### Determining the effect of haemodialysis initiation on serum (1–3)-β-D glucan

The effect of HD initiation on endotoxin and BG levels was explored in a separate cohort of pre-dialysis patients with CKD 5 (Table 4). Twenty-nine had full data and were analysed. Serum BG levels taken in the 6 months following dialysis initiation were significantly higher than those taken prior to initiation (26 vs. 41 pg/mL, *p* = 0.002). There were minimal increases in inflammatory markers post-initiation – significant only for TNF-α. Pre-dialysis, all patients had undetectable endotoxin. Following haemodialysis initiation, one patient tested positive for endotoxin only.

**Table 4:** Patient characteristics with endotoxin, (1–3)-β-D glucan, inflammatory cytokines, and symptoms pre- and post- initiation of haemodialysis

Parameter (***n*** = 29)		Value	
Age (year)		56 ± 16	
Male gender (%)		69	
Charlson Comorbidity Index		5 [3–5]	
Weight (kg)		87 ± 17	
	**Before HD**	**Post HD initiation**	***p*-value**
**Sample time (months)**	0 [0.28]	2.8 [2.3]	–
**BG (pg/mL)**	26 [17]	41 [22.5]	**0.002**
**IL-6 (pg/mL)**	5.9 [9.7]	7.3 [7.8]	NS
**TNF-α (pg/mL)**	19.4 [7.5]	21.9 [7.1]	**0.008**
**CRP (mg/L)**	5 [10.7]	5.6 [9.8]	NS
**Endotoxin (EU/mL)** **(without BG blockade)**	0 [0.032]	0 [0.026]	NS
**Detectable endotoxemia** **(without BG blockade) [%]**	31 (9/29)	28 (8/29)	NS
**Detectable endotoxemia** **(with BG blockade) [%]**	0 (0/29)	3.4 (1/29)	NS

For abbreviations see text. NS – p > 0.1. BG blocking buffer consists of highly concentrated carboxymethylated curdlan. Applying this buffer to the LAL assay the blocks the factor G pathway of the assay preventing any false activation by any circulating (1–3)-β-D glucan that may be present in the sample

## Discussion

We found that blood BG levels increased progressively with advancing CKD stage. BG levels were highest in dialysis patients with around a quarter of HD patients having BG levels above the cut-off value of 80 pg/mL for diagnosis of fungaemia. Across the CKD group high BG levels were associated with increased levels of inflammatory biomarkers, independently of CKD group. There was a small but significant increase in BG levels post dialysis initiation though inflammatory markers changed little.

The source of elevated BG in kidney disease is uncertain. BG are large molecules varying from tens to thousands kD. They are not significantly removed by dialysis and modern dialyser membranes do not influence blood levels [23]. Levels were inversely related to residual kidney function in dialysis patients although there was no relationship to either Kt/V or use of HDF, and though slightly higher levels were observed after HD initiation, it is unlikely that loss of renal function contributed. Previous studies have not found a significant effect of modern dialysis membranes on BG levels, however it is important to rule out the effect of membrane composition potentially leading to elevated BG levels and in this regard in-vitro studies may be useful. The metabolism of BG in humans is poorly understood, but in animal models BG is primarily removed by liver Kupffer cells, which comprise the major phagocytic activity of the mononuclear phagocyte system (MPS) [24]. Following intraperitoneal injection, the majority of BGs are distributed in organs prominent in the MPS, especially the liver [11, 25] and metabolised by oxidative degradation [12]. There are few data on Kupffer cell function in CKD, though MPS dysfunction has been described. Defects of immunity including defective phagocytic function, impaired maturation of monocytes and monocyte-derived dendritic cells have been reported [26–28]. Elevated blood BG levels detected in our population could relate to dysfunction of the MPS in advanced CKD.

Since the prevalence of high BG levels (> 80 pg/ml) were highest in HD patients who are inherently at increased risk of intra-dialytic and intestinal hypotension, the gut is another potential source of elevated BG. Although we were unable to detect a difference between our high-risk and low-risk HD patients this may have been due to the small sample size or that our high risk HD patients were not experiencing significantly worse intra-dialytic hypotension compared to low-risk patients. Advanced CKD may impair intestinal barrier function and result in translocation of bacterial products into the circulation - perhaps exacerbated by intradialytic gut hypo-perfusion [13, 29, 30]. Similarly, intestinal mucosal barrier damage and translocation has been described in end-stage kidney disease. Translocation of BG from the gut has been described and BG levels have been proposed as a marker of intestinal permeability [31–33]. There is a possible role for increased intestinal permeability in increasing systemic BG levels which may contribute to chronic inflammation. Similarly, the appearance of endotoxemia in advanced liver disease is thought to result from gut translocation together with MPS dysfunction [34]. True endotoxemia accounted for 5% of our HD cohort, a much lower prevalence than that reported in most other studies [35] though more in keeping with the clinical state of participants. Our study has a number of limitations. CKD sub-groups were not well matched so we cannot discount factors other than kidney function and treatment modality contributing to the observed differences in BG levels. The Fungitell assay is a biological assay which has inherent limitations [36]. It is well-validated though in many studies in other patient groups as a pan-fungal marker [37]. Reports of its accuracy in patients with CKD are more limited. It may be though that the threshold of > 80 pg/ml for the diagnosis of fungaemia needs to be revised in dialysis patients and comparison of BG levels in those with and without fungal infections in dialysis patients would be informative. In addition the numbers of patients with true endotoxaemia were too small to permit valid comparisons with other groups.

## Conclusion

This is the first study to investigate the relationship between renal function, blood BG levels and inflammation. Our findings show that blood concentration of BG progressively increased with advancing CKD and were highest in those on dialysis. There was a significant association of BG with inflammation – a poor prognostic marker in dialysis patients. Mechanisms of high BG levels are unknown but a combination of increased translocation from the gut and reduced clearance in the context of MPS dysfunction may contribute. High BG levels were also associated with inflammation, independently of CKD stage. BGs may have an important clinical role in patients with advanced kidney disease, particularly in dialysis patients.

## Data Availability

all data generated or analysed during this study are included in this published article.

## Abbreviations

BG: (1–3)-β-D glucans
HD: Haemodialysis
LAL: Limulus Amoebocyte Lysate assay
PD: Peritoneal dialysis
CKD: Chronic kidney disease
eGFR: Estimated glomerular filtration rate
IL-6: Interleukin-6
TNF-α: Tumour necrosis factor alfa
CV: Coefficient of variation
KRU: Residual urea clearance
HDF: Haemodiafiltration
CCI: Charlson Co-morbidity index
MPS: Mononuclear phagocyte system

## References

[1] Bashir KMI, Choi JS. Clinical and Physiological Perspectives of beta-Glucans: The Past, Present, and Future. Int J Mol Sci. 2017;18(9). 10.3390/ijms18091906. 10.3390/ijms18091906 PMC5618555 28872611

[2] Camilli G, Tabouret G, Quintin J (2018). The complexity of fungal beta-Glucan in health and disease: effects on the mononuclear phagocyte system. Front Immunol.

[3] Theel ES, Doern CD (2013). Beta-D-glucan testing is important for diagnosis of invasive fungal infections. J Clin Microbiol.

[4] Kambayashi J, Yokota M, Sakon M (1991). A novel endotoxin-specific assay by turbidimetry with Limulus amoebocyte lysate containing beta-glucan. J Biochem Biophys Methods.

[5] Wong J, Zhang Y, Patidar A, Vilar E, Finkelman M, Farrington K (2016). Is Endotoxemia in stable hemodialysis patients an Artefact? Limitations of the Limulus Amebocyte lysate assay and role of (1-->3)-beta-D Glucan. PLoS One.

[6] Kato A, Takita T, Furuhashi M, Takahashi T, Maruyama Y, Hishida A (2001). Elevation of blood (1-->3)-beta-D-glucan concentrations in hemodialysis patients. Nephron..

[7] Shendi AM, Davies N, Davenport A (2018). Systemic endotoxin in peritoneal Dialysis patients. Perit Dial Int.

[8] Pearson FC, Bohon J, Lee W (1984). Characterization of Limulus amoebocyte lysate-reactive material from hollow-fiber dialyzers. Appl Environ Microbiol.

[9] Kanda H, Kubo K, Hamasaki K (2001). Influence of various hemodialysis membranes on the plasma (1-->3)-beta-D-glucan level. Kidney Int.

[10] Prattes J, Schneditz D, Pruller F (2017). 1,3-ss-d-Glucan testing is highly specific in patients undergoing dialysis treatment. J Infect.

[11] Yoshida M, Roth RI, Grunfeld C, Feingold KR, Levin J (1997). Pharmacokinetics, biological effects, and distribution of (1-->3)-beta-D-glucan in blood and organs in rabbits. Mediat Inflamm.

[12] Nono I, Ohno N, Masuda A, Oikawa S, Yadomae T (1991). Oxidative degradation of an antitumor (1-3)-beta-D-glucan, grifolan. J Pharmacobiodyn.

[13] March DS, Graham-Brown MP, Stover CM, Bishop NC, Burton JO (2017). Intestinal barrier disturbances in Haemodialysis patients: mechanisms, consequences, and therapeutic options. Biomed Res Int.

[14] Jefferies HJ, Crowley LE, Harrison LE (2014). Circulating endotoxaemia and frequent haemodialysis schedules. Nephron Clin Pract.

[15] Goncalves S, Pecoits-Filho R, Perreto S (2006). Associations between renal function, volume status and endotoxaemia in chronic kidney disease patients. Nephrol Dial Transplant.

[16] Sulahian A, Porcher R, Bergeron A (2014). Use and limits of (1-3)-beta-d-glucan assay (Fungitell), compared to galactomannan determination (Platelia Aspergillus), for diagnosis of invasive aspergillosis. J Clin Microbiol.

[17] Wong J, Jeraj H, Vilar E, Viljoen A, Farrington K (2015). Endotoxin detection in end-stage kidney disease. J Clin Pathol.

[18] Wong J, Davies N, Jeraj H, Vilar E, Viljoen A, Farrington K (2016). A comparative study of blood endotoxin detection in haemodialysis patients. J Inflamm (Lond).

[19] AoCC (2011). Fungitell Assay: instruction for use.

[20] Petraitiene R, Petraitis V, Hope WW (2008). Cerebrospinal fluid and plasma (1-->3)-beta-D-glucan as surrogate markers for detection and monitoring of therapeutic response in experimental hematogenous Candida meningoencephalitis. Antimicrob Agents Chemother.

[21] Odabasi Z, Mattiuzzi G, Estey E (2004). Beta-D-glucan as a diagnostic adjunct for invasive fungal infections: validation, cutoff development, and performance in patients with acute myelogenous leukemia and myelodysplastic syndrome. Clin Infect Dis.

[22] He S, Hang JP, Zhang L, Wang F, Zhang DC, Gong FH (2015). A systematic review and meta-analysis of diagnostic accuracy of serum 1,3-beta-D-glucan for invasive fungal infection: focus on cutoff levels. J Microbiol Immunol Infect.

[23] Prattes J, Schilcher G, Krause R (2015). Reliability of serum 1,3-beta-D-glucan assay in patients undergoing renal replacement therapy: a review of the literature. Mycoses..

[24] Dixon LJ, Barnes M, Tang H, Pritchard MT, Nagy LE (2013). Kupffer cells in the liver. Compr Physiol.

[25] Suda M, Ohno N, Hashimoto T, Koizumi K, Adachi Y, Yadomae T (1996). Kupffer cells play important roles in the metabolic degradation of a soluble anti-tumor (1-->3)-beta-D-glucan, SSG, in mice. FEMS Immunol Med Microbiol.

[26] Verkade MA, van Druningen CJ, Vaessen LM, Hesselink DA, Weimar W, Betjes MG (2007). Functional impairment of monocyte-derived dendritic cells in patients with severe chronic kidney disease. Nephrol Dial Transplant.

[27] Lim WH, Kireta S, Leedham E, Russ GR, Coates PT (2007). Uremia impairs monocyte and monocyte-derived dendritic cell function in hemodialysis patients. Kidney Int.

[28] Kato S, Chmielewski M, Honda H (2008). Aspects of immune dysfunction in end-stage renal disease. Clin J Am Soc Nephrol.

[29] Vaziri ND, Goshtasbi N, Yuan J (2012). Uremic plasma impairs barrier function and depletes the tight junction protein constituents of intestinal epithelium. Am J Nephrol.

[30] Wang F, Jiang H, Shi K, Ren Y, Zhang P, Cheng S (2012). Gut bacterial translocation is associated with microinflammation in end-stage renal disease patients. Nephrology (Carlton).

[31] Leelahavanichkul A, Worasilchai N, Wannalerdsakun S (2016). Gastrointestinal Leakage Detected by Serum (1-->3)-beta-D-Glucan in Mouse Models and a Pilot Study in Patients with Sepsis. Shock (Augusta, Ga).

[32] Yang AM, Inamine T, Hochrath K (2017). Intestinal fungi contribute to development of alcoholic liver disease. J Clin Invest.

[33] Issara-Amphorn J, Surawut S, Worasilchai N (2018). The synergy of endotoxin and (1-->3)-beta-D-Glucan, from gut translocation, worsens Sepsis severity in a lupus model of fc gamma receptor IIb-deficient mice. J Innate Immun.

[34] Wang L, Llorente C, Hartmann P, Yang AM, Chen P, Schnabl B (2015). Methods to determine intestinal permeability and bacterial translocation during liver disease. J Immunol Methods.

[35] Wong J, Vilar E, Farrington K (2015). Endotoxemia in end-stage kidney disease. Semin Dial.

[36] Cohen J (2000). The detection and interpretation of endotoxaemia. Intensive Care Med.

[37] Theel ES, Jespersen DJ, Iqbal S (2013). Detection of (1, 3)-beta-D-glucan in bronchoalveolar lavage and serum samples collected from immunocompromised hosts. Mycopathologia..

